# Insight into the role of PIKK family members and NF-кB in DNAdamage-induced senescence and senescence-associated secretory phenotype of colon cancer cells

**DOI:** 10.1038/s41419-017-0069-5

**Published:** 2018-01-19

**Authors:** Anna Strzeszewska, Olga Alster, Grażyna Mosieniak, Agata Ciolko, Ewa Sikora

**Affiliations:** Laboratory of the Molecular Bases of Ageing, Nencki Institute of Experimental Biology, Polish Academy of Sciences, Warsaw, Poland

## Abstract

Senescence of cancer cells is an important outcome of treatment of many cancer types. Cell senescence is a permanent cell cycle arrest induced by stress conditions, including DNA damage. DNA damage activates DNA damage response (DDR), which involves members of the phosphatidylinositol 3-kinase-related kinase (PIKK) superfamily: protein kinases ATM, ATR, and DNA-PKcs. The so-far collected data indicate that ATM, with its downstream targets CHK2, p53, and p21, is the key protein involved in DDR-dependent senescence. It was also documented that the so-called senescence-associated secretory phenotype-SASP relies on ATM/CHK2, and not on p53 signaling. Moreover, genotoxic agents used in cancer treatment can activate NF-κB, which also induces transcription of SASP genes. In this paper, we have studied the involvement of three PIKK family members in colon cancer cell senescence and connection between DNA-damage-induced senescence and NF-κB-regulated SASP in p53-proficient and p53-deficient colon cancer cells treated with doxorubicin. We showed that doxorubicin induced cell senescence in both p53+/+ and p53−/− HCT116 cells, proving that this process is p53-independent. Senescence was successfully abrogated by a PIKK inhibitor, caffeine, or by simultaneous silencing of three PIKKs by specific siRNAs. By silencing individual members of PIKK family and analyzing common markers of senescence, the level of p21 and SA-β-Gal activity, we came to the conclusion that ATR kinase is crucial for the onset of senescence as, in contrast to ATM and DNA-PKsc, it could not be fully substituted by other PIKKs. Moreover, we showed that in case of silencing the three PIKKs, there was no SASP reduction accompanying the decrease in the level of p21 and SA-β-Gal (Senescence-Associated-β-Galactosidase) activity; whereas knocking down the NF-κB component, p65, abrogated SASP, but did not affect other markers of senescence, proving that DNA damage regulated senescence independently and NF-κB evoked SASP.

## Introduction

Senescence of cancer cells is an important outcome in treatment of cancers—especially those resistant to apoptosis in response to many chemotherapeutic agents. Cytostatic doses of agents which are less harmful for patients can be used in senescence-inducing therapy^1^. Cell senescence is a cell growth inhibition state, which arises due to telomere shortening (normal cells) or stress-induced cell cycle arrest (normal and cancer cells). Generally two signaling pathways, namely p16/Rb and p53/p21 are involved in the process of senescence^2^; however, cancer cells in which these pathways are disrupted are still prone to DNA-damage-induced cell senescence^3,4^. Senescence of cancer cells in vitro has been shown by many groups including our own^5–7^ and the number of publications showing induction of cancer cell senescence upon treatment with anticancer agents with DNA-damaging activity is constantly increasing^8^.

Double strand breaks (DSBs) activate the DNA damage response which involves ATM and ATR protein kinases, members of the phosphatidylinositol 3-kinase-related kinase (PIKK) superfamily. Another member of the PIKK family is the catalytic subunit of the DNA-dependent protein kinase (DNA-PKcs). Nonetheless, so-far collected data point to ATM, with its downstream targets CHK2, p53, and p21, as a key protein involved in DNA damage response^9^ and DNA-damage-induced senescence^10^. Interestingly, it was shown that the senescence-associated secretory phenotype (SASP) requires ATM/CHK2, but not p53 signaling^11^.

Genotoxic agents used in cancer treatment, such as ionizing radiation and topoisomerase I and II inhibitors (for example doxorubicin), can also activate the NF-κB pathway^12^. Thus, it cannot be excluded that NF-κB regulation might be involved in senescence of cancer cells. Especially since NF-κB activates transcription of SASP genes^13^.

NF-кB is an ubiquitously expressed family of transcription factors. In mammals, there are five members of the NF-кB/Rel family. The most abundant form of NF-кB is a heterodimer of p50 and p65 and the term NF-кB is often used to refer to this complex. In non-stimulated cells, NF-кB is sequestered in the cytoplasm in an inactive form through interaction with the IкB inhibitory proteins. In a canonical way, upon stimulation of cells by diverse cell stresses, the main member of IкB family, IкBα, is phosphorylated on two specific serine residues by a kinase (IKK) complex, which marks it for polyubiquitination. The degradation of IкBα by the proteasome leads to a rapid translocation of NF-кB to the nucleus, where it activates transcription from a wide variety of promoters, including that of its own inhibitor IкBα. The IKK complex contains two catalytic subunits and a regulatory subunit, NEMO^14^.

Recently it has been shown that senescence relays on NF-кB, as 65 of the upregulated and 26 of the downregulated genes in conditionally immortalized human fibroblasts are downstream targets of this transcription factor^15^. Others demonstrated that NF-kB controls both cell-autonomous and non-cell-autonomous aspects of the senescence program and identified a tumor-suppressive function of NF-kB that contributes to the outcome of cancer therapy^16^. However, the role of NF-кB in cell senescence still remains controversial^17^.

In this paper, we investigated whether PIKKs and NF-кB signaling pathways are involved in DNA-damage-induced senescence and SASP of colon cancer cells, especially since an interaction between ATM and NF-кB signaling was discovered^12^. To this end, we induced senescence in p53-proficient and p53-deficient cancer cells with a known anticancer DNA-damaging agent, doxorubicin.

## Results

### Doxorubicin induced DNA-damage-dependent senescence of p53+/+ and p53−/− cells

**Fig. 1:**
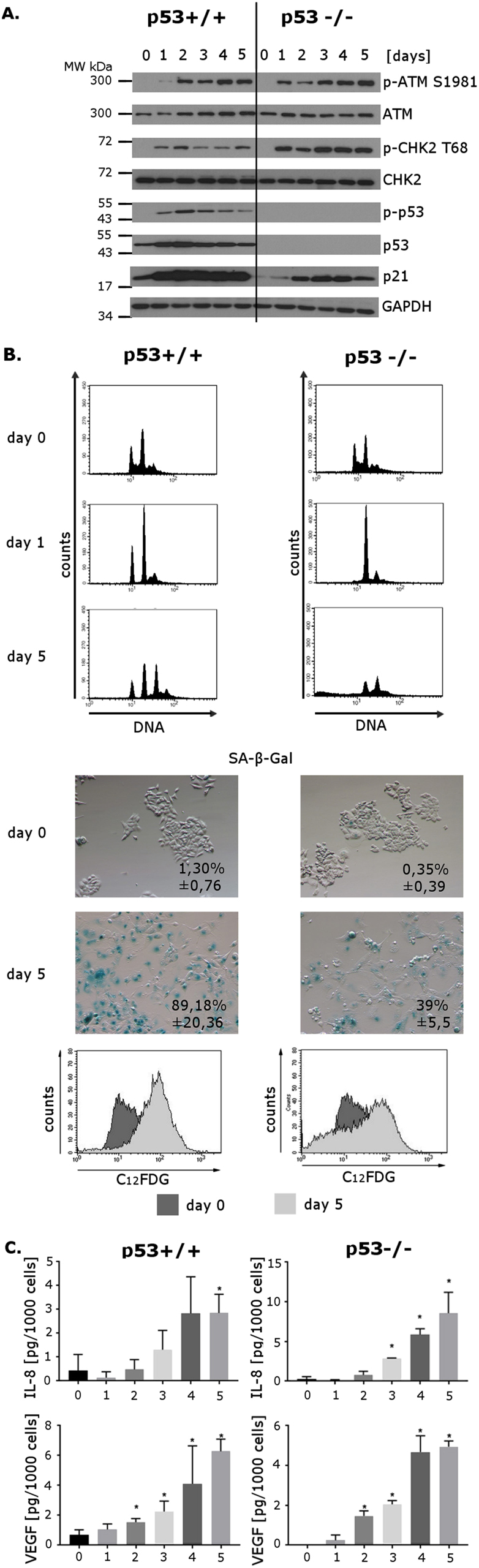
Doxorubicin-induced senescence of HCT116 p53+/+ and p53−/− cells Numbers denote days of culture with doxorubicin. **a** The levels of key DDR proteins in untreated cells (day 0) and on the consecutive days (1–5) after doxorubicin treatment, analyzed by Western blotting. GAPDH was used as a loading control. **b** Upper panel: DNA content analyzed by flow cytometry, in untreated cells and on subsequent days after doxorubicin (100 nM) treatment (days 1 and 5). Representative histograms from one of three independent experiments. Central panel: SA-β-Gal activity (cytochemical staining) in untreated cells and in cells treated with doxorubicin for 5 days (HCT116 p53+/+ as well as HCT116 p53−/−). Representative images from one of three independent experiments. Numbers depict percentage of SA-β-Gal-positive cells ± standard deviation. Magnification 100×. Lower panel: SA-β-Gal activity (cytometric analysis) in untreated cells and in cells treated with doxorubicin for 5 days (HCT116 p53+/+ as well as HCT116 p53−/−). Representative histograms from one of three independent experiments, illustrating C_12_FDG fluorescence. The dark gray histogram represents control cells, the light gray histogram represents cells which were cultured in the presence of doxorubicin **c**. Amount of IL-8 (upper panel) and VEGF (lower panel) secreted by HCT116 p53+/+ (left) and p53−/− cells (right) on consecutive days of doxorubicin treatment (depicted in numbers from 0 to 5) measured with ELISA. Bars represent mean value of three independent experiments. Error bars represent standard deviation

We have performed our studies using p53+/+ and p53−/− HCT116 cells (derived from colorectal carcinoma) as well as H1299 cells (derived from non-small-cell lung carcinoma). H1299 cells have a homozygous partial deletion of the p53 gene and lack the expression of its protein. Cells were cultured in the presence of a low, cytostatic (100 nM) dose of doxorubicin (dox) continuously for 5 days and every 24 h we analyzed the level of ATM/CHK2/p53/p21, DNA content, and SA-β-Gal activity. The increased levels of p-ATM (Ser 1981) and p-CHK2 (Thr 68) were observed in all cell types upon dox treatment (Fig. 1a and Supplementary Fig. 1A). Additionally, in case of the p53+/+ HCT116 cells, an increased level of p-p53 (Ser 15) was detected (Fig. 1a). The level of p-CHK2 in HCT116 p53−/− cells was more pronounced than that found in p53+/+ cells. Both proteins, p53 and CHK2, are ATM substrates; thus we hypothesized that in p53-deficient cells, higher phosphorylation of CHK2 could be due to the lack of the other target for ATM. Activation of the DNA damage response (DDR) pathway was accompanied by the appearance of such markers of senescence as increased SA-β-Gal activity and cell cycle arrest (Fig. 1b and Supplementary Fig. 1B). Interestingly, in p53-proficient cells, the cell cycle arrest was observed in the G1 and G2 phases, while in p53-deficient cells it was observed in the G2 phase. In all cell lines, we observed a time-dependent increase in the level of the cell cycle inhibitor, p21 (Fig. 1a and Supplementary Fig. 1A), a widely used marker of senescence, which is classically transactivated by p53; however, it can also be activated in a p53-independent manner^18^. Since the key DDR protein, namely ATM, but not p53, was shown to be necessary for SASP of non-cancer cells^11^, we have checked the amount of two secreted factors typical for SASP in both p53+/+ and p53−/− HCT116 cells. As expected, in both types of cells we detected a substantial increase in the secretion of two SASP components, namely IL-8 and VEGF secretion upon dox treatment (Fig. 1c).

### Downregulation of *RELA* protected cells from the establishment of a doxorubicin-induced secretory phenotype but not from senescence

**Fig. 2:**
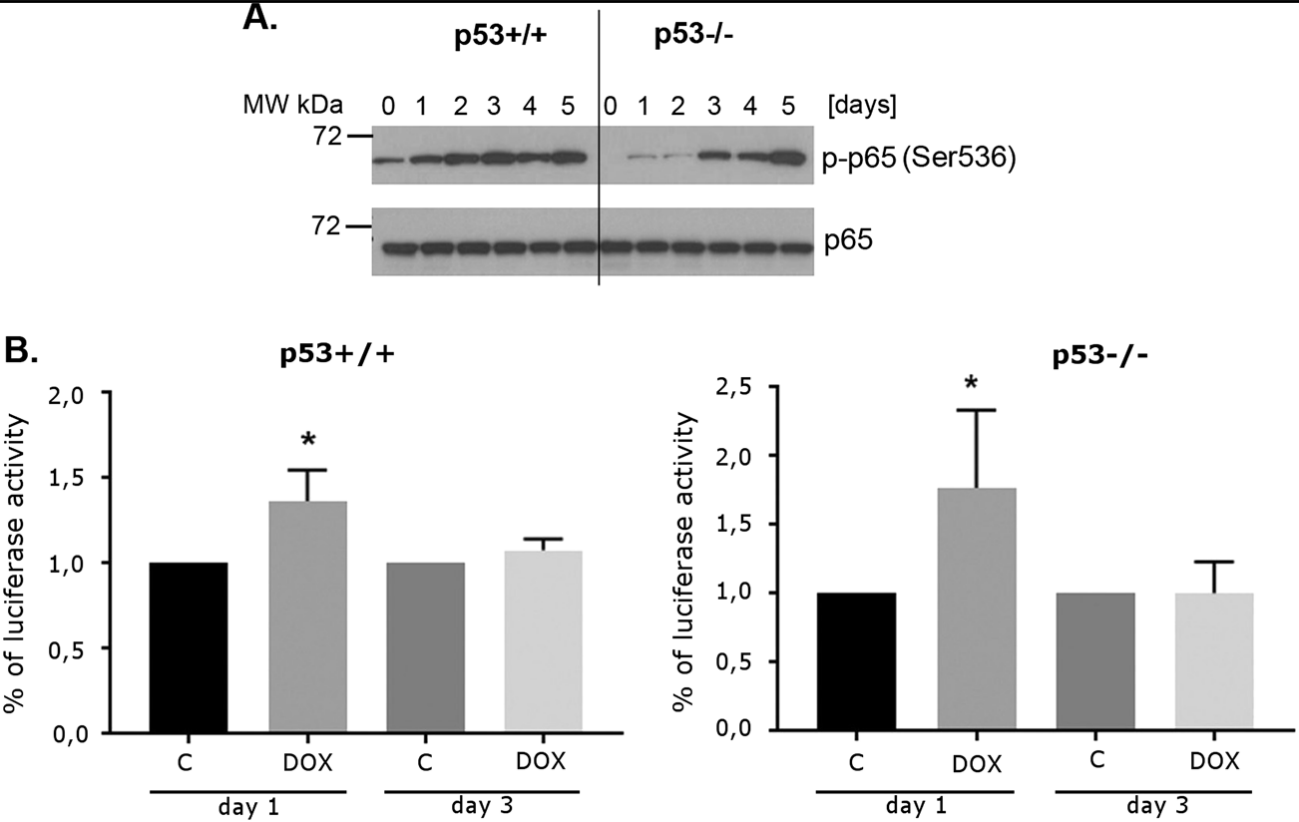
The activity of NF-κB transcription factor in HCT116 cells (p53+/+ and p53−/−) treated with doxorubicin **a** The level of phosphorylated (active form) of p65 subunit of NF-κB transcription factor complex was estimated using Western blot analysis in dox-treated HCT116 cells (p53+/+ and p53−/−). Representative blots are shown. **b** NF-κB activity was measured after 1 and 3 days of doxorubicin treatment using a reporter assay. Normalized luciferase activity (firefly/renilla) was calculated. Fold change of normalized luciferase activity between control (C) and dox- treated cells (DOX) is presented on graphs. Mean values of three independent experiments. Bars represent standard deviation

As the majority of SASP components are products of genes transactivated by transcription factor NF-κB^11^, we were interested whether the main component of NF-κB, p65 (encoded by *RELA* gene), is indispensable for SASP in HCT116 cells treated with dox. Western blot revealed rapid increase in the level of p-p65 upon dox treatment. It is known that p65 phosphorylation on Ser 536 increases its function of transactivation^19^. Also, luciferase assay showed a transient increase of NF-κB transcriptional activity in cells treated with doxorubicin (as compared to control cells) (Fig. 2a, b).

**Fig. 3:**
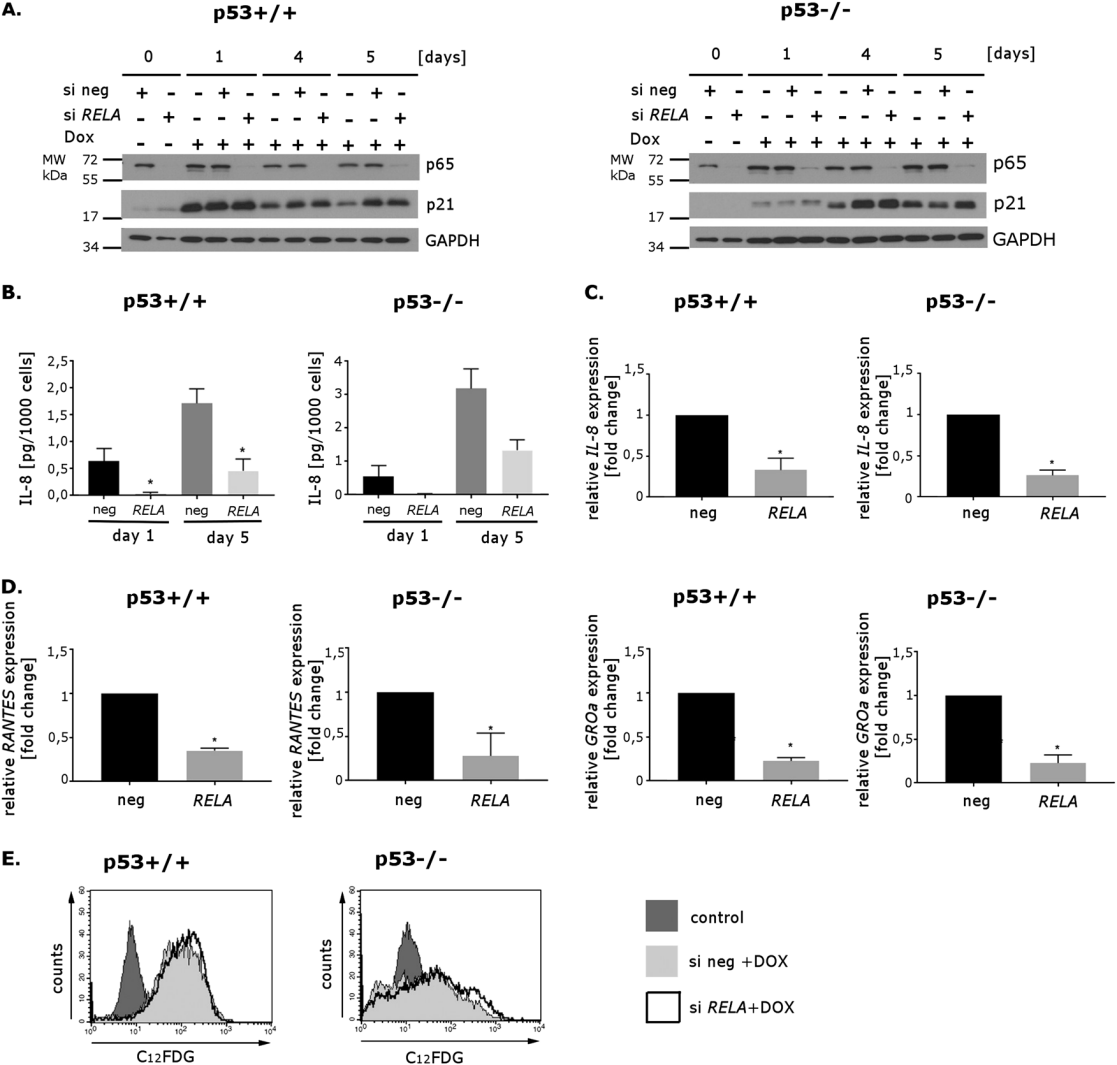
The influence of downregulation of gene *RELA* (encoding NF-κB p65 subunit) on senescence of HCT116 p53+/+ and p53−/− cells HCT116 p53+/+ and p53−/− cells were transfected with negative siRNA or *RELA* siRNA (30 nM). Two days after transfection, the cells were treated with doxorubicin (100 nM) for 5 days. **a** Whole cell extracts were prepared at indicated time points after treatment with doxorubicin. The level of p65 and p21 proteins was estimated by Western blotting; GAPDH was used as a loading control. **b** ELISA analysis of of IL-8 secreted by HCT116 p53+/+ (left graph) and p53−/− cells (right graph) transfected with negative siRNA or with siRNA targeting *RELA* on days 1 and 5 of doxorubicin treatment; mean value of three independent experiments. Error bars represent standard deviation. **c** RT-PCR analysis of expression of IL-8 in cells transfected either with negative siRNA or with *RELA* siRNA, treated with doxorubicin for 5 days. Results were normalized to the level of GAPDH mRNA. Mean values of three independent experiments. Error bars represent standard deviation. **d** RT-PCR analysis of expression of selected NF-κB-regulated genes (*RANTES* and *GROα*) in cells transfected either with negative siRNA or with *RELA* siRNA, treated with doxorubicin for 5 days. Results were normalized to the level of GAPDH mRNA. Mean values of three independent experiments. Error bars represent standard deviation. **e** SA-β-Gal activity measured by flow cytometry. Representative histograms illustrating C_12_FDG fluorescence in HCT116 p53+/+ (left) and p53−/− (right) cells

To investigate the role of NF-κB in senescence, we transfected the HCT116 p53+/+ and p53−/− cells with either negative siRNA or *RELA* siRNA. Two days after transfection, the cells were treated with dox and cultured in the presence of this agent for 5 days. The p65 protein was undetectable till the last day of the experiment (Fig. 3a). As expected, in cells with silenced *RELA*, we observed lower amount of secreted IL-8 (Fig. 3b) in comparison with control cells (transfected with negative RNA). These results were confirmed by real-time polymerase chain reaction (RT-PCR) measurements (Fig. 3c). Also the mRNA levels of other proteins characteristic for the secretory phenotype, such as RANTES and GROα, were decreased by *RELA* siRNA proving the NF-кB transcriptional activity in dox-induced SASP (Fig. 3d). Next, we have checked SA-β-Gal activity and unexpectedly we found that it was comparable in cells transfected either with negative siRNA or *RELA* specific one (Fig. 3e) indicating that NF-κB is not involved in cell senescence, but is necessary for SASP.

### Caffeine protects HCT116 cells from senescence

**Fig. 4:**
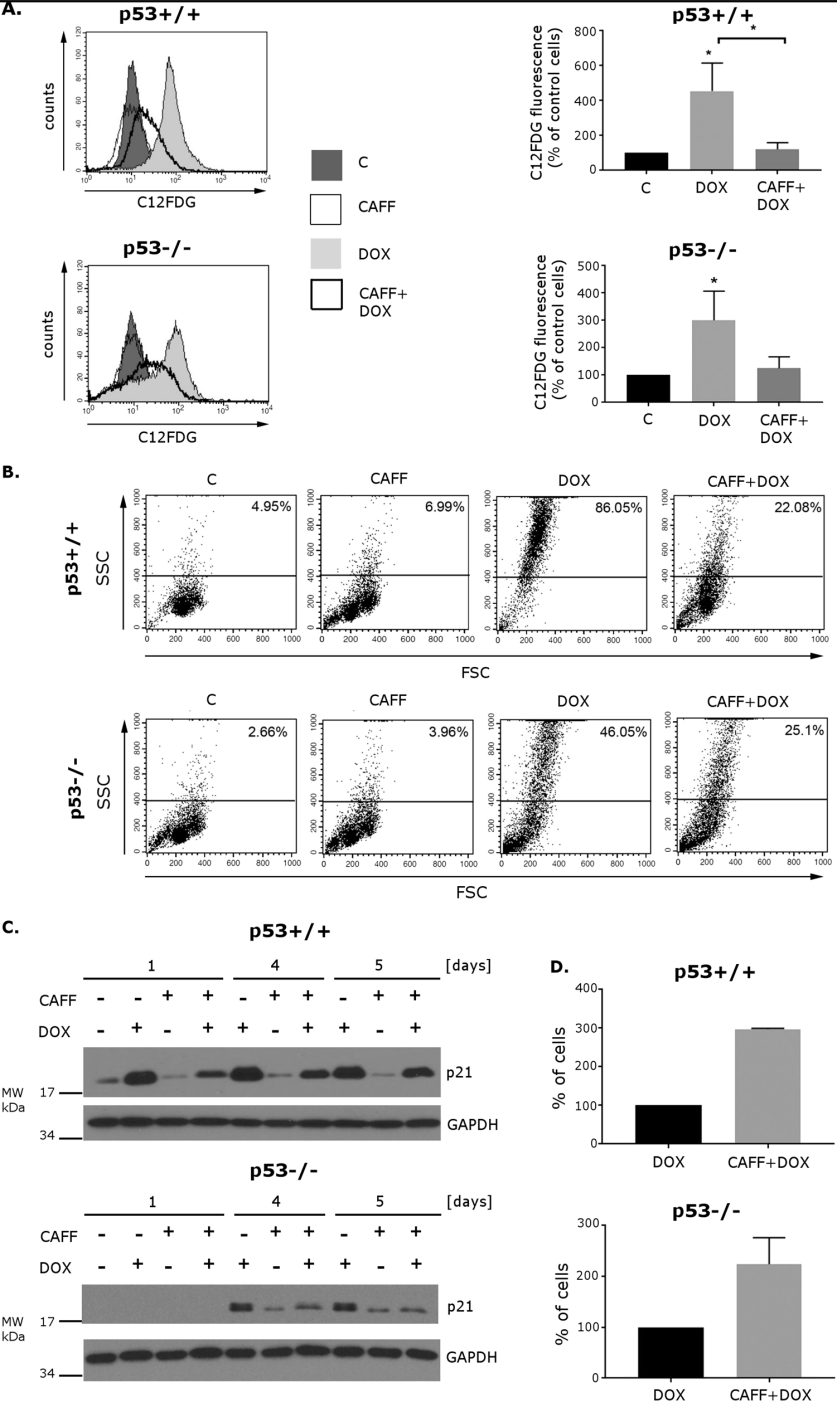
The effect of caffeine on doxorubicin-induced senescence Cells were pretreated for 2 h with 2 mM caffeine and cultured in the presence of 100 nM doxorubicin for 5 days. **a** SA-β-Gal activity measured by flow cytometry. Representative histograms illustrating C_12_FDG fluorescence in HCT116 p53+/+ (upper panel) and p53−/− cells (lower panel). Geometrical mean values of C_12_FDG fluorescence measured in three independent experiments are summarized in graphs **b** Representative dot-plots illustrating changes in cell granularity. Number on each dot-plot indicates the percentage of cells with increased granularity. Data were analyzed using the CellQuest software **c** The level of p21 protein in untreated cells and on the consecutive days after doxorubicin/caffeine/doxorubicin+caffeine treatment, analyzed by Western blotting. GAPDH was used as a loading control. **d** Number of living cells after pretreatment with caffeine and treatment with doxorubicin for 5 days relative to the number of doxorubicin-only treated cells (mean value of three independent experiments). Error bars represent standard deviation

To confirm the role of ATM in senescence of p53+/+ and p53−/− HCT116 cells, we used a widely known inhibitor of the PIKKs—caffeine^20^, and analyzed different markers of cell senescence (Fig. 4). The HCT116 cells were pretreated for 2 h with 2 mM caffeine and afterward treated with 100 nM dox and cultured in the presence of this agent for 5 days. Higher SA-β-Gal activity and cell granularity, measured by flow cytometry, were observed in dox-treated cells than in control (untreated) ones, while pretreatment with caffeine substantially decreased the activity of SA-β-Gal and cell granularity (Fig. 4a, b) and preserved the cell proliferation capabilities (Fig. 4d). Furthermore, we observed that pretreatment with caffeine led to a decrease in the level of p21 in p53+/+ as well as in p53−/− cells (Fig. 4c) indicating that caffeine-treated cells can overcome the growth arrest caused by doxorubicin.

Based on the obtained results, we can conclude that PIKKs are involved in senescence of both p53+/+ and p53−/− HCT116 cells.

### ATR is indispensable for senescence of both p53+/+ and p53−/− HCT116 cells treated with doxorubicin

**Fig. 5:**
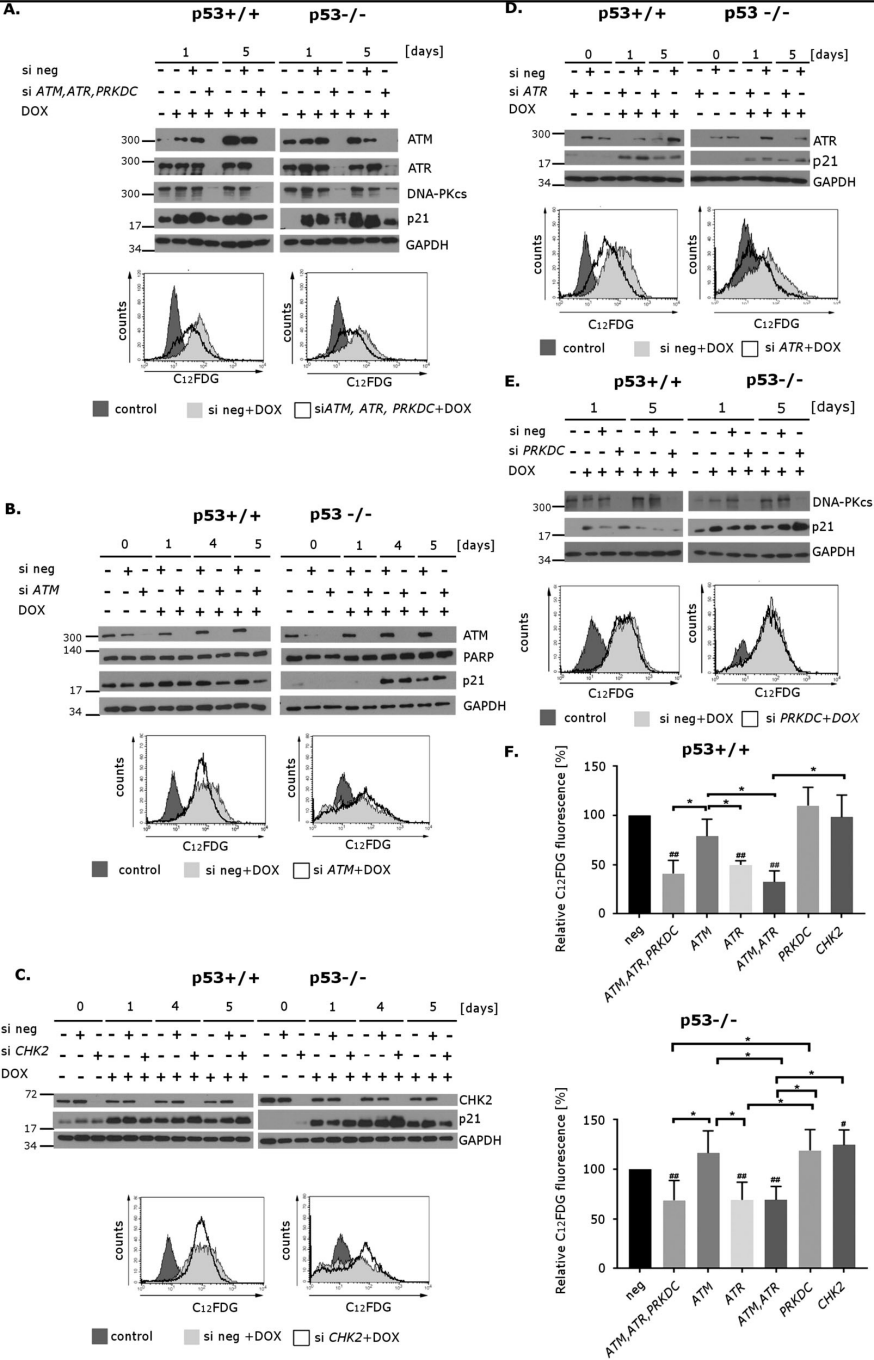
The role of PIKKs in doxorubicin-induced senescence of HCT116 p53+/+ and p53−/− cells Cells were transfected with negative siRNA or siRNA depicted in the picture (30 nM). Two days after transfection, the cells were treated with doxorubicin (100 nM) for 5 days. For Western blot analysis, whole cell extracts were prepared at indicated time points after treatment with doxorubicin. For SA-β-Gal analysis, samples were collected after 5 days of doxorubicin treatment. **a** Triple silencing  of *ATM*, *ATR*, and *PRKDC*. Upper panel: protein levels (ATM, ATR, DNA-PKcs, p21) were evaluated using Western blotting. GAPDH was used as a loading control. Lower panel: SA-β-Gal activity measured by flow cytometry. Representative histograms illustrating C_12_FDG fluorescence in HCT116 p53+/+ and p53−/− **b**.  Silencing  of *ATM*. Upper panel: protein levels (ATM, PARP, p21) were evaluated using Western blotting. GAPDH was used as a loading control. Lower panel: SA-β-Gal activity measured by flow cytometry. Representative histograms illustrating C_12_FDG fluorescence in HCT116 p53+/+ and p53−/− cells **c**. Silencing of *CHK2*. Upper panel: protein levels (CHK2, p21) were evaluated using Western blotting. GAPDH was used as a loading control. Lower panel: SA-β-Gal activity measured by flow cytometry. Representative histograms illustrating C_12_FDG fluorescence in HCT116 p53+/+ and p53−/− **d** Silencing of *ATR*. Upper panel: protein levels (ATR, p21) were evaluated using Western blotting. GAPDH was used as a loading control. Lower panel: SA-β-Gal activity measured by flow cytometry. Representative histograms illustrating C_12_FDG fluorescence in HCT116 p53+/+ and p53−/− **e.** Silencing of *PRKDC*. Upper panel: protein levels (DNA-PKcs, p21) were evaluated using Western blotting. GAPDH was used as a loading control. Lower panel: SA-β-Gal activity measured by flow cytometry. Representative histograms illustrating C_12_FDG fluorescence in HCT116 p53+/+ and p53−/− cells **f**. Summarized relative C_12_FDG fluorescence of cells treated with siRNA targeting indicated genes—single or in combinations (relative to fluorescence of cells treated with negative siRNA). Mean values of at least three independent experiments are shown. Error bars represent standard deviation. Hashtags indicate statistically significant differences between given data points and data obtained for negative siRNA. Asterisks indicate statistically significant differences between pairs marked on the graphs

To systematically study the role of PIKKs in p53-dependent and p53-independent cell senescence, before treatment of HCT116 cells with dox, we transfected them with specific siRNA silencing the expression of three PIKKs (ATM, ATR, and DNA-PKcs). We confirmed, by Western blotting, successful silencing of all these kinases on days 1 and 5 (Fig. 5a). Like in the case of caffeine, we observed lower level of p21 and significantly lower activity of SA-β-Gal (Fig. 5a, f) in cells transfected with specific siRNA, which dropped to about 40% and 60% of the level observed in their counterparts treated with negative siRNA (p53+/+ and p53−/− cells, respectively; SA-β-Gal activity measured on day 5 of dox treatment).

Next, we investigated whether these kinases act as redundant systems of DNA-damage-induced senescence or whether each of them performs a distinct function. First we silenced *ATM*, which has been described as the main sensor upon DSB induction^21^. Unexpectedly, we did not observe a decrease in p21 level or in SA-β-Gal activity in p53−/− cells, and only a slight p21 reduction and decrease in SA-β-Gal, which was not statistically significant, in p53+/+ cells (Fig. 5b, f). As CHK2 is the main substrate of ATM that becomes phosphorylated after dox treatment (Fig. 1a), and since previously CHK2 was proposed to be responsible for p53-independent senescence of cancer cells^18^, we silenced *CHK2* expression (Fig. 5c). However, we neither observed a lower level of p21 nor quantitatively lower activity of SA-β-Gal in cells transfected with CHK2 siRNA in comparison with those transfected with negative siRNA. This indicated that under the given experimental conditions, loss of ATM/CHK2 activity could be compensated by other PIKKs, which is consistent with previous studies on ATM-deficient cells^22–25^.

Next we silenced *ATR* expression. It appeared that this kinase has a more pronounced effect than ATM on senescence of both p53+/+ and p53−/− HCT116 cells, as we observed statistically significant decrease in SA-β-Gal activity of about 50% in p53+/+ and a decrease of about 30% in p53−/− cells transfected with specific siRNA as compared to cells transfected with negative siRNA (accompanied with a slight change in p21 level) (Fig. 5d, f).

In Fig. 5f, showing a compilation of SA-β-Gal activity of all variants of gene silencing presented in Fig. 5a–e, there can also be found SA-β-Gal activity in cells with simultaneously silenced *ATM* and *ATR*. These data point to ATR as a crucial kinase for induction of senescent phenotype, as only silencing of ATR expression resulted in statistically significant decrease in SA-β-Gal activity—which seemed to be further decreased by downregulation of ATM in case of p53+/+ cells. Also, silencing of ATR, but not ATM, reduced cell granularity measured by flow cytometry (Supplementary Fig. 2A), which is a good indicator of cell senescence^26^. BrdU incorporation assay shows that cell proliferation is improved in case of ATR silencing only in HCT116 p53+/+ cells (Supplementary Fig. 2B). It cannot be excluded that HCT116 p53−/− cells need more time for recovery.

Finally, we silenced expression of DNA-PKcs (*PRKDC*), and observed no effect on p21 level or SA-β-Gal activity either in p53+/+ or p53−/− cells (Fig. 5e, f). We can thus assume that this kinase is not indispensable in dox-induced senescence of HCT116 cells, when ATM and ATR are present. Nevertheless, we cannot totally reject the role of DNA-PKcs in senescence, as in triple knockdown experiment we observed that only after downregulating simultaneously ATM, ATR, and DNA-PKcs, the level of p21 was clearly reduced, which was not the case when single knockdowns were performed (in case of SA-β-Gal activity, this effect was not as evident, Fig. 5f). We postulate a phenomenon of mutual substitution between PIKKs in signal transduction to explain these observations and point to a novel, crucial role of ATR in doxorubicin-induced senescence.

### PIKKs are not necessary for SASP

**Fig. 6:**
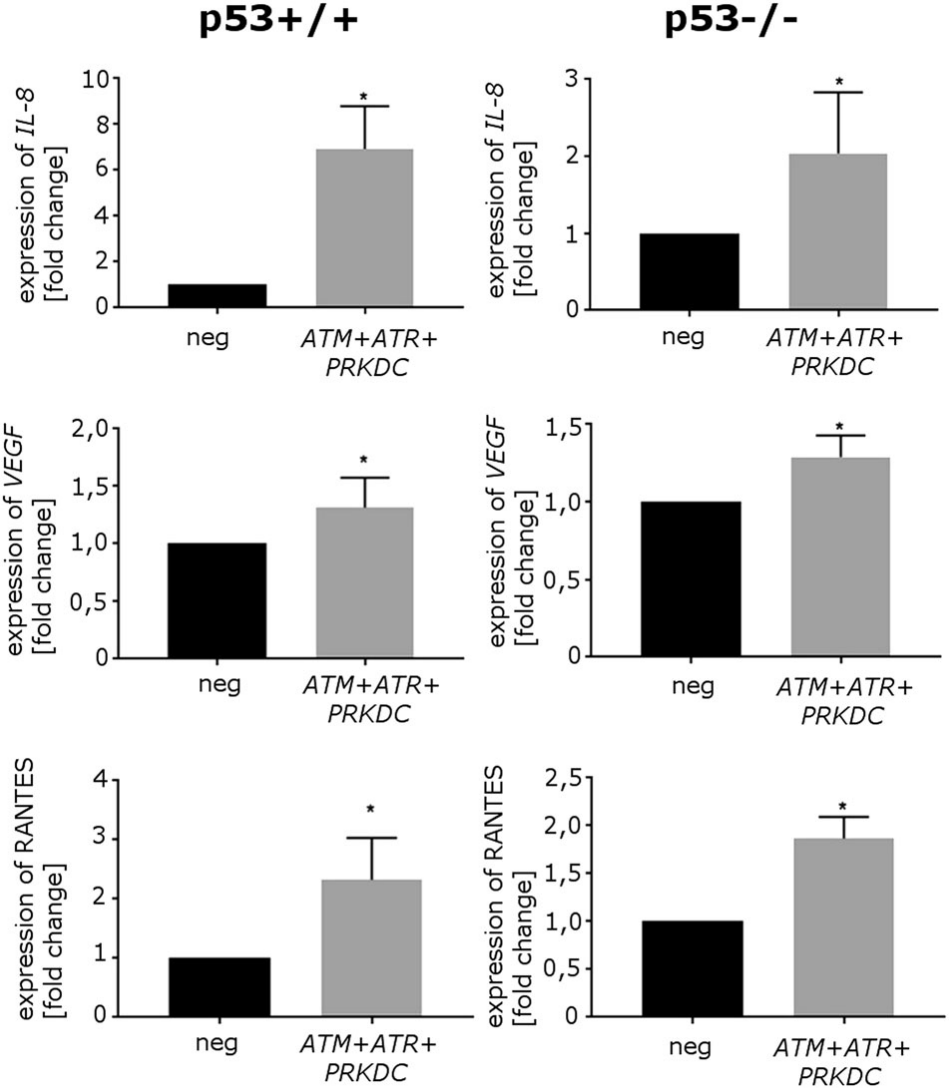
The impact of simultaneous ATM, ATR, and DNA-PKc downregulation on senescence-associated secretory phenotype RT-PCR analysis of expression of selected senescence-associated secretory phenotype genes (*IL-8*, *VEGF*, *RANTES*) in cells transfected either with negative siRNA or with combination of siRNAs targeting *ATM*, *ATR*, and *PRKDC*, treated with doxorubicin for 5 days. Results were normalized to the level of GAPDH mRNA. Normalized relative expression levels are shown (mean value of three independent experiments). Bars represent standard deviation

Results obtained clearly show that all the investigated PIKKs (ATM, ATR, and DNA-PKcs) are required for p21 level increase in response to dox treatment both in p53+/+ and p53−/− HCT116 cells, but ATR seems to play a major role in the induction of SA-β-Gal activity. Moreover, NF-кB is necessary for the secretory phenotype but not for enhancement of p21 level and SA-β-Gal activity. To finally asses the distinctiveness of these two phenotypes, we checked the secretory activity in cells with all three PIKKs’ genes silenced. Figure 6 shows that in cells deficient in active kinases, the expression of secreted factors was fully preserved as revealed by IL-8, VEGF, and RANTES mRNA levels in adherent (alive) cells assessed by RT-PCR. Moreover, it seems that silencing of PIKKs genes led to even higher expression of these factors in comparison with cells transfected with negative siRNAs (Fig. 6).

## Discussion

Recently DDR, which leads to cell senescence, took on a great importance in anticancer therapy as irradiation and many chemotherapeutic agents operate via DNA damage in cancer cells^27^. Although it is commonly believed that constant activation of DDR has a causative role in cell senescence^10^, just recently it has been shown that, indeed, DNA damage can alone induce cell senescence^28^. Accordingly, we showed that a DNA-damaging agent, doxorubicin, induced senescence in cancer cells and that caffeine, a common inhibitor of PIKKs (ATM, ATR, and DNA-PKc) involved in DDR^29^, abrogated senescence of both p53+/+ and p53−/− HCT116 cells. Interestingly, cells which escaped DNA-damage-induced cell cycle arrest due to caffeine treatment, appeared to proliferate rather than undergo cell death, contrary to the results obtained by Crescenzi et al.^30^ and Vavrova et al.^31^. However, in support of our results, caffeine has been shown before to protect from ionizing radiation or genotoxic treatment^32^ or to diminish the cytotoxic effects of doxorubicin, ellipticine, and mitoxantrone^33^.

As caffeine inhibits all three PIKKs, we were interested which one is crucial in dox-induced senescence of HCT116 cells or whether they can substitute for each other upon DNA damage, especially in cells lacking their main substrate, namely p53. The *ATM* and *TP53* genes, whose protein products are crucial for DDR, are commonly mutated tumor-suppressor genes. In cells and tumors that lack a functional p53 pathway, ATM inactivation is sufficient to globally sensitize them to killing by genotoxic chemotherapy, demonstrating a synthetic lethal interaction between these two tumor-suppressor genes^34^. However, we observed that even in p53-deficient HCT116 cells silencing of *ATM*, or downstream *CHK2*, did not induce cell death upon low-dose doxorubicin treatment (not shown). ATM deficiency led to a slightly lower SA-β-Gal activity upon dox treatment in p53+/+, but not p53−/− cells. Silencing of *CHK2* neither influenced cell senescence in p53+/+ nor in p53−/− cells, suggesting that ATM/CHK2 activity can be successfully compensated by that of ATR and/or DNA-PKcs.

DNA-PKc is induced in response to doxorubicin and plays a key role in the repair of DSBs by non-homologous end-joining (NHEJ). Inhibition of DNA-PKcs has been considered as a novel and attractive approach to decrease resistance to therapeutically induced DSBs^35–37^, but data concerning its role in senescence are scarce. Rocourt et al. showed that pretreatment of normal human diploid fibroblasts with DNA-PKcs kinase inhibitor NU 7026 suppressed selenium-induced senescence response^38^ and Salminen et al. discovered that this kinase was downregulated in senescent fibroblasts, which could probably contribute to accumulation of DNA damage during aging^39^. Recently, it has been shown that loss of DNA-PKcs function led to hyperactivation of ATM and amplification of the p53 response, sensitizing cells to damage-induced senescence^40^. Our results indirectly support the presence of such mechanism. Although we did not check the activity of ATM upon DNA-PKcs gene silencing, we did not observe significant differences in SA-β-Gal activity between dox-treated ATM- or DNA-PKcs-depleted p53+/+ and p53 −/− cells and control cells (transfected with negative siRNA). Interestingly, Azad et al. observed that senescence of irradiated human cancer cells was accelerated when DNA-PKc was inhibited^41^.

Our next goal was to dissect the role of ATR in the signaling network operating during cancer cell senescence. Several papers showed that there was a strict connection between ATM and ATR upon DSBs. Communication between ATM and ATR enables the cell to respond to DSBs and inhibition of DNA synthesis with highly coordinated outputs^42–45^.

Although doxorubicin is believed to induce primarily DSBs while ATR is a single-strand breaks (SSBs) sensor kinase, ATR activation in response to doxorubicin was observed before^46^. In our study, downregulation of the level of ATR did counteract senescence induction both in p53-proficient and in p53-deficient HCT116 cells. Downregulating both kinases (ATM and ATR) affected cellular senescence comparably to silencing only ATR in p53−/− cells, whereas in p53+/+ cells downregulation of ATM together with ATR seemed to affect SA-β-Gal activity slightly more than downregulation of ATR alone.

This could suggest that in p53+/+ cells, ATM and ATR depletion can have an additive effect. Similar results supporting this conclusion were obtained in the case of silencing of all three genes. The open question is how DNA damage signal is transduced to p21 in cell lacking p53 expression. Cell senescence has been shown in many cancers lacking p53, but it seems that in that case the main role of cell cycle inhibition belongs to p16^47^. However, in HCT116 cells, its expression is repressed due to promoter methylation^48^. A p53-independent role of CHK2 in p21 induction and senescence has been described^18^. It seems that this is not the case of HCT116 cells because even though we observed even higher CHK2 phosphorylation in p53−/− than p53+/+ cells, the silencing of *CHK2* had no effect on dox-induced senescence in both cell lines. As cell senescence is a very complex trait and beside DDR, there can be other signaling pathways leading directly or indirectly to p21-dependent cell cycle arrest reviewed in ^49^. This issue requires further elucidation.

Altogether, our study indicates that beside the central role of ATM kinase in DDR and senescence that was established years ago, ATR could have a vital functional relevance in these processes. Further studies could provide new insights into regulation of cell fate upon genotoxic insult, especially in p53-deficient cells.

It seems that HCT116 colon cancer cells have very efficient mechanisms preserving their ability to undergo senescence upon genotoxic treatment. In terms of efficiency of anticancer treatment, this could be either beneficial or detrimental. Considering senescence as a desirable outcome of anticancer treatment^27^, the cell ability to activate the senescence program optionally by one of the two DNA damage sensors (ATM or ATR) is very promising. On the other hand, it is believed that cancer cell senescence can be the reason of cancer cells resistance to anticancer treatment^50^. We showed previously that not only doxorubicin^6,7^ but also other anticancer drugs used in clinic for colon cancer treatment, such as methotrexate, arrested cell proliferation^51^. Moreover, reversibility of cancer cell senescence is becoming pointed out (reviewed by Sikora et al^8^).

Another undesirable feature of senescent cancer cells is their SASP, which can influence the microenvironment and reinforce senescence of neighboring cells^52^. Accordingly, we were interested in the mechanisms of SASP upon DNA damage. It is well documented that the role of ATM in senescence is not limited to the cell cycle arrest, but that its activation (together with CHK2 and NBS1) is essential for establishing and maintaining the expression of several SASP proteins, including IL-8^11^. In response to genotoxic insult, ATM can activate NF-kB, which is believed to be the factor stimulating the appearance of SASP^53^. We confirmed the role of NF-kB in SASP, as downregulation of NF-kB subunit p65 (RelA) resulted in a reduced level of secreted IL-8 and lower levels of mRNAs encoding IL-8, RANTES, and GROα. Interestingly, attenuated SASP was not coupled to a decrease in p21 level and SA-β-Gal activity, proving that NF-kB is necessary for establishing SASP independently of the cell cycle arrest. Moreover, we observed that the level of IL-8 mRNA was not decreased in cells with silenced PIKKs expression. Intriguingly, we even observed increased levels of IL-8 and other SASP components (VEGF and RANTES) mRNAs upon PIKK genes silencing. It cannot be excluded that in HCT116 cells SASP is independent of all PIKKs, contrary to what has been broadly documented for other types of cells (reviewed by Sabatel et al^54^). The growing body of evidence shows that alternative upstream signaling pathways can be involved in NF-kB activation leading to the establishment of the secretory phenotype. Namely, activation of NF-kB could be triggered by p38MAPK^55^ and mTOR^56^. Recently Ferrand et al. identified 33 kinases whose constitutive expression induced senescence and SASP components. Focusing on some of those kinases, showing the strongest pro-senescence effects, they observed that all of them induced expression of SASP-component genes through activation of an NF-κB-dependent transcriptional program^57^. Moreover, there are several studies showing the strict connection between dysfunctional mitochondria in senescent cells, which produce reactive oxygen species (ROS) thus activating SASP^58–60^. Although it seems that ROS are necessary and sufficient to activate NF-кB, they can in a feedback loop induce DDR, thus participating in the establishment of the senescence phenotype^58^. Doxorubicin as an topoisomerase II inhibitor causes DNA damage, but it also, independently, increases ROS level^61^. Thus blocking DDR evoked by doxorubicin and following senescence, we could still preserve doxorubicin ROS-producing activity followed by NF-кB activation and SASP. Previously we showed that ROS scavenging in dox-treated HCT116 cells did not protect against senescence, but we did not check SASP in this experimental setting^6^. Nonetheless, our results presented in this study revealed that p65 silencing did not abrogate senescence. This suggests that in HCT116 cells induction of senescence and induction of the secretory phenotypes are distinct events. Moreover, we want to stress that cancer cell senescence, even if is characterized by many features of senescence of normal cells, to some extent can possess different properties.

## Materials and methods

### Cell lines/Cell culture and treatments

HCT116 p53+/+ and p53−/− cells were cultured in McCoy’s medium supplemented with 10% FBS (fetal bovine serum) and antibiotics. The cells were cultured at a density of 10^4^ cells/cm^2^. A day before transfection, cells were seeded at a density of 5 × 10^3^ cells/cm^2^. H1299 cells were cultured in RPMI-1640 (Roswell Park Memorial Institute-1640) medium supplemented with 10% FBS, L-glutamine, and antibiotics (streptomycin and penicillin). Cells were cultured at a density of 5 × 10^3^ /cm^2^. A day before transfection, the cells were seeded at a density of 4 × 10^3^ cells/cm^2^. To induce premature senescence, all three cell lines were treated with 100 nM doxorubicin (Sigma Aldrich, Poznan, Poland). In order to analyze the involvement of PIKK family kinases in dox-induced senescence, we used a specific inhibitor, i.e., caffeine (Sigma Aldrich). The cells were pretreated for 2 h with caffeine and dox was added to the medium afterward.

### DNA content and cell cycle analysis

For DNA analysis, the cells were fixed in 70% ethanol and stained with PI solution (3.8 mM sodium citrate, 500 µg/ml RNAse, 50 µg/ml PI in PBS). All agents were purchased from Sigma Aldrich (Poznan, Poland). DNA content was assessed using flow cytometry and analyzed with the CellQuest Software. A total of 10,000 events were collected per sample (FACSCalibur, Becton Dickinson, Warsaw, Poland).

### Western blotting analysis

Whole cell protein extracts were prepared using the SLB buffer (50 mM Tris-HCl pH 6.8, 10% glycerol, 2% SDS). The amount of protein was measured using the BCA method. Equal amounts of proteins (20 µg) were separated electrophorectically in 8%, 12%, or 15% SDS-polyacrylamide gels and afterwards transferred to nitrocellulose membranes. Membranes were blocked in 5% non-fat milk dissolved in TBS containing 0.1% Tween-20 (Sigma Aldrich, Poznan, Poland) for 1 h at room temperature (RT) and incubated with one of the primary monoclonal or polyclonal antibodies: anti-ATM (1:500), anti-p-ATM Ser 1981 (1:1000), anti-DNA-PKcs (1:2000), anti-p-DNA-PKcs Ser 2056 (1:1000), H2AX (1:500) and anti-p-H2AX Ser 139 (1:1000) (Abcam, Cambridge, UK), anti-p53 (DO-1) (1:500), anti-p21(C-19) (1:500) (Santa Cruz Biotechnology Inc., Dallas, Texas, USA), anti-p-p53 Ser 15, anti-Chk2, anti-p-Chk2 Thr 68, anti-p65 and anti-p-p65 Ser 536 (Cell Signaling, Lab-JOT Ltd., Warsaw, Poland), anti-PARP1 (1:1000) (Becton Dickinson, Diag-med, Warsaw, Poland), and anti-GAPDH (1:50000) (Merck Millipore, Warsaw, Poland). The proteins were detected with appropriate secondary antibodies conjugated with horseradish peroxidase and ECL reagents (Thermo Scientific), according to the manufacturer’s protocol.

### Silencing of the *RELA*, *CHK2*, *ATM*, *ATR*, and *PRKDC* genes/Transfection assays

To downregulate *RELA*, *CHK2*, *ATM*, *ATR*, or *PRKDC* expression, the HCT116 p53+/+ and p53−/− cells were seeded in 6- or 12-well plates (50 × 10^3^ or 20 × 10^3^ cells per well, respectively) and transfected with 30 nM siRNA (against *RELA*, *CHK2*, *ATM*, *ATR*, *PRKDC*, or negative) (Life Technologies, Warsaw, Poland) using Lipofectamine 2000 (Life Technologies, Warsaw, Poland). Transfection was performed according to the manufacturer’s protocol. Twenty-four hours after transfection, the medium was replaced with a fresh one and cells were cultured for 5 days in the presence of doxorubicin (100 nM) (Sigma Aldrich, Poznan, Poland).

### Cytochemical detection of SA-β-Gal

Detection of SA-β-Gal was performed according to Dimri et al^62^. Briefly, cells were fixed with 2% formaldehyde, 0.2% glutaraldehyde in PBS, washed, and exposed overnight at 37 °C to a solution containing 1 mg/ml 5-bromo-4-chloro-3-indolyl-b-D-galactopyranoside, 5 mM potassium ferrocyanide, 5 mM potassium ferrycyanide, 150 mM NaCl, 2 mM MgCl_2_, and 0.02 M phosphate buffer, pH 6.0. All of the used agents were purchased from Sigma Aldrich (Poznan, Poland). Photos were taken in transmitted light using the Nikon Eclipse Ti-U fluorescent microscope and Nikon Digital Sight DS-U3 camera (Nikon, Warsaw, Poland).

### Flow cytometric detection of SA-β-Gal

SA-β-gal activity was measured by flow cytometry, using the fluorogenic substrate 5-dodecanoylaminofluorescein di-beta-d-galactopyranoside (C_12_FDG) as described previously^7^. Cells were incubated with 33 μM C_12_FDG (LifeTechnologies) in 37 °C for 30 min. Afterward, cells were washed with PBS and analyzed immediately with BD FACSCalibur flow cytometer (Beckton Dickinson, BD Bioscience). Data were analyzed using the CellQuestPro software. SA-β-gal activity was estimated using the mean fluorescence intensity of the cell population.

### Bromodeoxyuridine incorporation assay

For DNA synthesis assay, cells were grown on coverslips and bromodeoxyuridine (BrdU, Sigma—Aldrich) was added to the medium (10 µM) and cells were cultured for 24 h. Thereafter, cells were fixed in cold 70% ethanol and stored at −20 °C at least overnight. For BrdU detection, primary antibody against BrdU (Becton Dickinson) and a secondary Alexa 488-conjugated IgG antibody (Becton Dickinson) were used. Cells were observed under a fluorescence microscope (Nikon, Tokyo, Japan) with the use of 450–490 nm excitation wavelength and photos were taken using the Evolutions VF digital CCD camera (MediaCybernetics). At least 200 cells per experiment were analyzed and the percentage of BrdU-positive cells were calculated.

### Quantitative RT-PCR

Quantitative RT-PCR was used to quantify the mRNAs for IL-8, VEGF, RANTES, and GRO-α with expression of GAPDH used as endogenous control. Total RNA was isolated from cells with RNeasy Micro Kit (Qiagen) according to the manufacturer’s instruction. First-strand cDNA was synthesized using 0.5 μg of total RNA. cDNA was mixed with SYBR Green Master Mix (Applied Biosystems, Thermo Fisher Scientific) and 1 μM primers for human IL-8 (forward: 5’-AGGGTTGCCAGATGCAATAC-3’, reverse: 5’-CCTTGGCCTCAATTTTGCTA-3’), human VEGF (forward: 5’-CGAGGGCCTGGAGTGTGT -3’, reverse: 5’-CGCATAATCTGCATGGTGATG-3’), human RANTES (forward: 5’-GAAGGA AGTCAGCATGCCTC-3’, reverse: 5’-AGCCGATTTTTCATGTTTGC-3’), human GRO-α (forward: 5’-GAACATCCAAAGTGTGAACGTGAAG-3’, reverse: 5’-TTCAGGAACAGCCACCAGTGAG-3’), and human GAPDH (forward: 5’-TGCACCACCAACTGCTTAGC-3’, reverse: 5’-GAGGGGCCATCCACAGTCTTC-3’). The reactions were performed with the use of either StepOnePlus ™ Real-Time PCR System (Thermo Fisher Scientific)—in the case of triple knockdown experiments (siRNA against *ATM*, *ATR*, and *PRKDC*)—or with 7500 Real-Time PCR System (Applied Biosystems) in the case of all other RT-PCR experiments. Results were analyzed using relative quantification—the ΔΔCt approximation method.

### Luciferase assay

To assess the activity of NF-κB, we used the Dual-Luciferase Reporter Assay System (Promega). Cells were seeded on 24-well plates for 24 h before transfection. Cells were transiently transfected with 0.5 μg of pGL4.32[luc2P/NF-κB-RE/Hygro] and 0.14 μg pRL-SV40 reference plasmid (Promega) as an internal control. Twenty-four hours post-transfection, cells were treated with 100 nM doxorubicin (control cells were left untreated). Luciferase activity was assessed after 24 h using a TD-20/20 luminometer (Turner Designs).

### ELISA assay

To assess the secretion of IL-8 and VEGF proteins culture medium was collected and subjected to analysis according to the manufacturer’s instructions (R&D Systems). Absorbance was measured using Tecan Sunrise instrument.

### Statistical analysis

Data were analyzed using GraphPad software. Data concerning RT-PCR experiments as well as IL-8 and VEGF secretion on consecutive days of doxorubicin treatment were analyzed using one-tailed Mann–Whitney *U* test. All other experiments were analyzed with two-tailed Mann–Whitney *U* test. Statistical significance is indicated in the pictures with asterisks and hashtags (*p*-value < 0.05 indicated with single mark, *p*-value < 0.01 indicated with double mark).

## Electronic supplementary material

Suplementary Figure 1

Suplementary Figure 2
