# Supplementary material for: Insight into the role of PIKK family members and NF-кB in DNAdamage-induced senescence and senescence-associated secretory phenotype of colon cancer cells
Source: Cell Death Dis. 2018 Jan 19;9(2):44. doi: 10.1038/s41419-017-0069-5 (PMC5833415; doi:10.1038/s41419-017-0069-5)
Supplement: Supplementary file 1 — Suplementary Figure 1 [file 41419_2017_69_MOESM1_ESM.pdf]

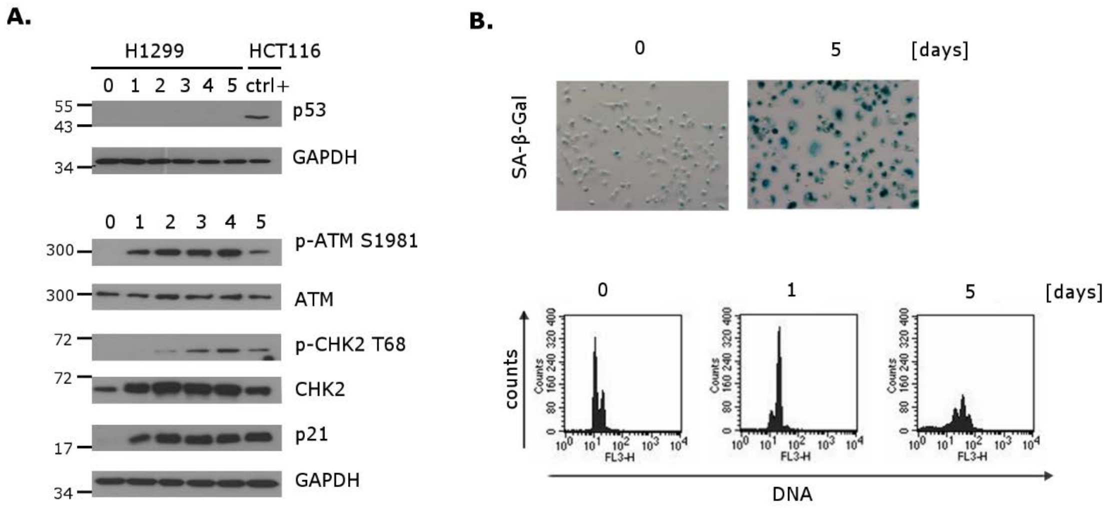

Supplementary Figure 1. Doxorubicin-induced senescence of H1299 cells. Numbers denote days of culture with doxorubicin (100 nM). **A. Upper panel:** The level of p53 analyzed by Western blotting in untreated and in doxorubicin treated cells. Cell lysate from HCT116 p53 +/+ cells was used as a positive control. **Lower panel:** The level of key DDR proteins after doxorubicin treatment analyzed by Western blotting. GAPDH was used as a loading control **B. Upper panel:** SA-β-Gal activity (analyzed by cytochemical staining) in untreated cells as well as in cells treated with doxorubicin for 5 days. Representative images from one of three independent experiments, magnification 100x. **Lower panel**: DNA content, analyzed by flow cytometry, in untreated cells and on subsequent days after doxorubicin treatment (day 1 and 5). Representative histograms from one of three independent experiments
