# Supplementary material for: Insight into the role of PIKK family members and NF-кB in DNAdamage-induced senescence and senescence-associated secretory phenotype of colon cancer cells
Source: Cell Death Dis. 2018 Jan 19;9(2):44. doi: 10.1038/s41419-017-0069-5 (PMC5833415; doi:10.1038/s41419-017-0069-5)
Supplement: Supplementary file 2 — Suplementary Figure 2 [file 41419_2017_69_MOESM2_ESM.pdf]

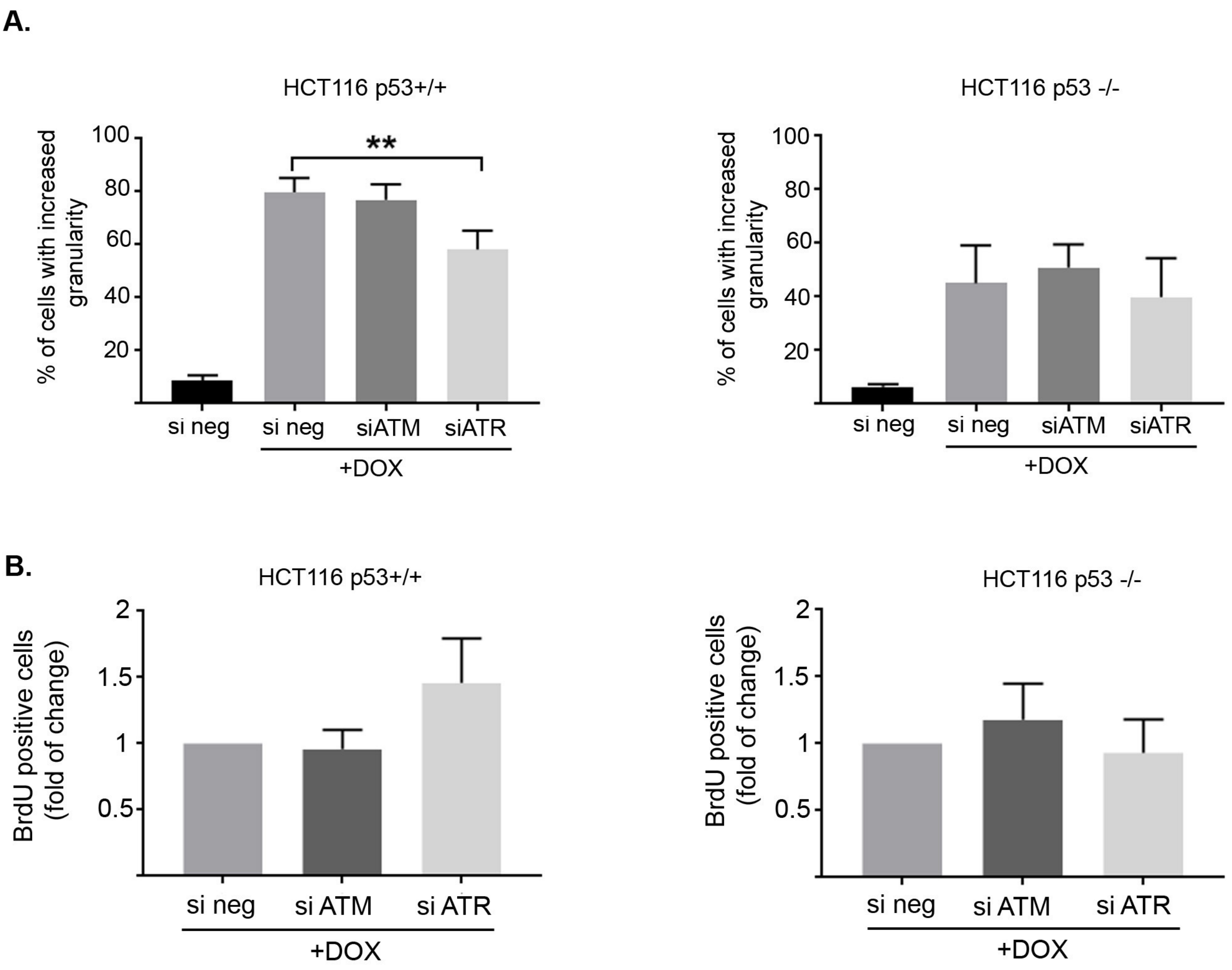

Spplementary Figure 2.
The impact of ATM and ATR silencing on marker of senescence (high granularity) and proliferation (BrdU incorporation).

Cells were transfected with negative siRNA or siRNA depicted in the picture (30nM). Two days after transfection the cells were treated with doxorubicin (100 nM) for five days. After 5 days of culture cells were analyzed by flow cytometry and the percentage of cells characterized by increased granularity (high SSC) were estimated using CellQuest software **(A)**. **B.** The number of BrdU incorporating cells were estimated in cells transfected with negative siRNA and siRNA targeting ATM or ATR three days after doxorubicin treatment.
